# Vagus nerve stimulation alleviates alveolar bone loss and inflammation in ligature-induced periodontitis rat model

**DOI:** 10.1590/1678-7757-2025-0322

**Published:** 2025-11-03

**Authors:** XIE Honghui, Ping HUANG, TAN Aihong, SU Zhijian, FU Ying

**Affiliations:** 1 Changsha Stomatological Hospital Department of Endodontics Hunan China Changsha Stomatological Hospital, Department of Endodontics, Hunan, China.; 2 Deyang People’s Hospital Department of Stomatology Sichuan China Deyang People’s Hospital, Department of Stomatology, Sichuan, China.; 3 Karamay Central Hospital The Second Medical College of Xinjiang Department of Stomatology Xinjiang Uygur Autonomous Region China Karamay Central Hospital, The Second Medical College of Xinjiang, Department of Stomatology, Xinjiang Uygur Autonomous Region, China.

**Keywords:** Vagus nerve stimulation, Periodontitis, Alveolar bone loss, Inflammation, α7-nAChR

## Abstract

**Objective:**

Vagus nerve stimulation (VNS) can inhibit inflammation in various diseases by activating the cholinergic anti-inflammatory pathway. However, whether VNS could attenuate periodontitis by activating α7-nicotinic acetylcholine receptor (α7-nAChR) remains unknown.

**Methodology:**

Ligature induction was utilized to establish the periodontitis rat model. Periodontal indices like bleeding, tooth mobility, and probing depth, were measured. Bone mineral density, trabecular thickness, and length of the cement-enamel junction to the alveolar bone crest were analyzed using micro-CT. Immunohistochemistry assessed bone morphology and inflammatory levels. Inflammatory cytokines were detected using enzyme-linked immunosorbent assay and quantitative reverse transcription polymerase chain reaction. For the *in vitro* inflammation model, RAW264.7 cells were stimulated with lipopolysaccharides and acetylcholine to study inflammatory responses.

**Results:**

VNS significantly improved periodontal health and reduced alveolar bone loss in periodontitis rats. VNS alleviated inflammation by suppressing pro-inflammatory cytokines, including IL-1β, IL-6, and IL-18, enhanced bone formation, and activated the cholinergic anti-inflammatory pathway, as evidenced by increased α7-nAChR expression. Additionally, acetylcholine activation of α7-nAChR in RAW264.7 cells inhibited pro-inflammatory responses and promoted anti-inflammatory responses.

**Conclusion:**

These findings suggest that VNS can effectively reduce inflammation and improve periodontal outcomes in periodontitis.

## Introduction

A common inflammatory condition in adults, periodontitis gradually damages the periodontal tissues, ultimately leading to tooth loss.^1,2^ Periodontal pathogens and their metabolic byproducts play a key role in modulating local and systemic immune responses, with the severity of periodontal disease being positively associated with age.^3^ Given the global aging population and the increasing number of individuals retaining their natural teeth, periodontal damage is expected to affect more people in the future.^4^ Local impacts of periodontal disease are well recognized, including gingival inflammation, bleeding upon probing, attachment loss, and increased tooth mobility.

Researchers believed that the nervous system controlled inflammatory responses solely through the hypothalamic-pituitary axis and neuroendocrine pathways.^5^ However, recent studies have identified a cholinergic anti-inflammatory pathway between the nervous and immune systems involving the vagus nerve and its neurotransmitter acetylcholine (ACh),^6^ which plays a crucial role in regulating inflammatory responses. The α7-nicotinic acetylcholine receptor (α7-nAChR) is a widely distributed receptor in the central nervous system and an integral component of the cholinergic anti-inflammatory pathway.^7^ Its activation can regulate the expression of pro-inflammatory mediators, thereby modulating local and systemic inflammation. The cholinergic anti-inflammatory pathway relies on ACh as a neurotransmitter which is synthesized in the neuronal cell body, transported along the axon to the terminal, and stored in synaptic vesicles.^8^ Upon release, Ach binds to α7-nAChRs on target cells to exert its effects. This pathway is widely distributed, with α7-nAChRs also found in peripheral tissues,^9^ suggesting their potential as a therapeutic target for periodontitis.

Although current treatments can help alleviate periodontitis symptoms, their effectiveness is often limited by long-term side effects and the temporary nature of their results.^10^ Exploring novel therapeutic strategies, particularly non-pharmacological interventions, is thus critical. In recent years, vagus nerve stimulation (VNS) has become a promising non-invasive treatment approach with significant anti-inflammatory effects across various disease models.^11,12^ VNS activates the cholinergic anti-inflammatory pathway, modulating immune responses and exerting anti-inflammatory and immune-regulatory effects.^13,14^ VNS initiates the cholinergic anti-inflammatory pathway by activating efferent vagal fibers, leading to ACh release at peripheral nerve terminals. ACh binds to α7-nAChRs expressed on immune cells such as macrophages, thereby significantly suppressing the production of pro-inflammatory cytokines, including TNF-α, IL-1β and IL-6, via inhibition of the nuclear factor-κB (NF-κB) signalling pathway.^15,16^ In parallel, VNS modulates systemic immune responses by regulating splenic immune cell activity via the vagus nerve–splenic nerve axis.^17,18^ This mechanism involves a synergistic interaction with the sympathetic nervous system.^19^ Notably, non-invasive transcutaneous auricular vagus nerve stimulation (taVNS) has also been shown to exert anti-inflammatory effects via this pathway.^20^ Experimental evidence indicates that this neuroimmune circuit effectively attenuates inflammation in models of rheumatoid arthritis, inflammatory bowel disease, and ischemia-reperfusion injury.^21,22^ The key molecular mediator (α7-nAChR) has thus emerged as a critical therapeutic target in treatments for neuroinflammatory disorders.^23^ Previous research has shown a close association between the cholinergic anti-inflammatory pathway and the anti-inflammatory effects of electroacupuncture (EA).^24^ Activation of the cholinergic anti-inflammatory pathway regulates the body’s inflammatory responses. This process involves the release of ACh from vagal nerve terminals which binds to α7-nAChRs on immune cells, leading to inflammation suppression.^25^ Studies have observed that EA can activate the JAK2/STAT3 signaling pathway mediated by α7-nAChR on macrophages, alleviating intestinal inflammation.^26^ Moreover, EA can mitigate dry eye disease inflammation by regulating the α7-nAChR/NF-κB signaling pathway.^27^ While these studies highlight the role of α7-nAChR in mediating EA-induced anti-inflammatory effects, it remains unclear whether direct VNS can achieve similar outcomes in periodontitis. In the present study, we employed direct cervical VNS using implanted electrodes to explore this possibility. Thus, our study developed a periodontitis rat model to explore the potential of VNS in activating α7-nAChR for treating periodontitis both *in vivo* and *in vitro*, and thus offer new insights and evidence for non-pharmacological treatments of periodontitis.

## Methodology

### Rat experiment

Male Sprague-Dawley rats, aged 6-8 weeks and weighing 200-250 g, were allowed one week to acclimate before being randomly assigned to three groups (n=8 per group): control, periodontitis, and periodontitis with VNS. All rats completed the experimental procedures without loss or exclusion. Following electrode implantation for cervical vagus nerve stimulation, the animals were placed in a heated chamber to recover and then returned to standard housing conditions. No adverse effects like persistent agitation, loss of appetite, or infection were observed. General behavior, activity levels, and food intake remained normal across all groups throughout the study period. As expected, rats in the periodontitis group exhibited gingival inflammation and bleeding, whereas those in the periodontitis with VNS group showed gradual symptom relief following intervention. Periodontitis was induced by anesthetizing the animals with 2% sodium pentobarbital and placing 0.2 mm orthodontic ligature wires around the cervical area of the bilateral maxillary first molars. The ligatures were inspected daily and replaced if dislodged, with the procedure continuing for 4 weeks. Animal studies were approved by Changsha Stomatological Hospital (#2024-05-27).

### VNS intervention

After inducing periodontitis, VNS was performed as previously described.^28^ Rats were anesthetized and positioned in the supine stance. The fur over the left cervical region was shaved, and a small incision was made along the left midline of the neck. Dissection of the skin and subcutaneous fascia exposed the cervical muscles, which were retracted to reveal the carotid sheath. The vagus nerve was carefully isolated from the carotid sheath using a blunt dissection technique. A nylon-coated wire was then wrapped around the vagus nerve, and the electrode was securely fixed in place. The wire was tunneled subcutaneously to the back of the neck, where it was anchored using sutures. Post-operatively, the animals were placed in a heated recovery chamber until fully conscious and then returned to their cages.

In the VNS group, the stimulation parameters were set to 1.0 mA, a pulse width of 0.5 ms, and a frequency of 30 Hz. Rats received daily stimulation for the first 14 days, each lasting 10 minutes, followed by alternate-day stimulation for the next 14 days, with each session lasting 10 minutes. This phased stimulation schedule was designed to provide intensive anti-inflammatory activation during the acute phase of inflammation, followed by a maintenance phase to sustain therapeutic effects while minimizing overstimulation or physiological adaptation, as supported by previous studies.^28^

### Periodontal analysis

After 28 days, periodontal health of the animals was assessed, focusing on changes in gingival color, shape, and texture. The following parameters were also measured:

Gingival Bleeding Index (BI): A periodontal probe was gently inserted into the gingival sulcus of the maxillary first molar, and the bleeding index was recorded using the Mazza scoring system.

Tooth Mobility (TM): The maxillary first molar was gently rocked in the buccolingual, mesiodistal, and vertical directions with forceps. Mobility was scored as follows: 0 = no mobility; 1 = mobility in the buccolingual direction; 2 = mobility in both the buccolingual and mesiodistal directions; 3 = mobility in all directions.

Probing Depth (PD): A blunt-ended probe measured the probing depth at six points around the maxillary first molar (mesiobuccal, distobuccal, and central), and the average depth was calculated.

### Micro-computed tomography (CT) analysis

The maxillary bones were fixed in 4% paraformaldehyde at 4°C for 2 days, then scanned using Micro-CT with parameters set to 70 kV, 200 mA, and 300 ms. 3D reconstruction of the alveolar bone was performed using IPL software. The region at the root bifurcation of the maxillary first molar was analyzed for bone mineral density (BMD), trabecular thickness (Tb.Th), and bone volume/total volume (BV/TV). ImageJ software measured the length from the cement-enamel junction (CEJ) to the alveolar bone crest (ABC) on both the mesial and distal aspects of the maxillary first molar, and the average was calculated.

### Cell culture

Frozen cells from the liquid nitrogen tank were quickly thawed in a pre-warmed 37 °C water bath. Once fully thawed, the cells were transferred to a 15 mL tube containing complete culture medium and centrifuged at 1500 rpm for 4 minutes. After removing the supernatant, fresh complete medium was added to resuspend the cells. The cell suspension was transferred to a culture dish with cell culture medium and maintained in a 37 °C incubator with 5% CO_2_.

### *In vitro* inflammation model

RAW264.7 macrophage cell line was culture in DMEM medium (Gibco, Grand Island, NY). The *in vitro* inflammation model was induced by treatment with 500 ng/mL lipopolysaccharides (LPS, eBioscience, CA, USA) to study an inflammatory response. The LPS-treated cells were incubated at 37°C for 24 hours. For Ach intervention, 1 μmol/L Ach chloride was added to the appropriate groups following LPS stimulation.

### Enzyme-linked immunosorbent sssay (ELISA)

Periodontal tissues were lysed in radioimmunoprecipitation assay buffer with a protease inhibitor and incubated with shaking for 1 hour at 4 °C. After centrifugation at 12,000 rpm for 10 minutes at 4 °C, the supernatant was collected and stored at -80 °C. Cytokine levels including IL-1β (Abcam, MA, USA), IL-18 (Abcam), and IL-6 (Abcam) were measured using ELISA kits according to the manufacturer’s instructions.

### Immunohistochemistry

Paraffin-embedded sections of periodontal tissues from the maxillary molar region—including gingival epithelium, connective tissue, periodontal ligament, and adjacent alveolar bone—were prepared for staining. The paraffin sections were preheated for 30 minutes and dewaxed with xylene. After hydration with graded ethanol, antigen retrieval was performed using pepsin for 15 minutes, followed by 1 x PBS washing. Endogenous peroxidase was blocked, and the sections were incubated with normal goat serum. Primary antibodies against ALP (1:250, Abcam, MA) and IL-1β (1:250, Abcam) were incubated overnight at 4°C. After washing, biotinylated goat anti-rabbit IgG and horseradish peroxidase-labeled streptavidin were added, followed by diaminobenzidine staining. The reaction was stopped with another wash, and sections were counterstained with hematoxylin, differentiated, and blued with ammonia water. The sections were then dehydrated, cleared, mounted, and observed under a microscope.

For quantification, 3-5 representative high-power fields (400×) were randomly selected from each section. ImageJ software (NIH, USA) analyzed DAB staining by applying the “Color Threshold” function to detect brown-positive areas. The percentage of positive staining area relative to total field area was calculated, and the average value across fields was used for each sample. Data were normalized to the control group for statistical analysis.

### Hematoxylin-eosin (H&E) staining

The fixed maxillary bones were decalcified in 15% EDTA for 1 month, followed by standard dehydration. After embedding in paraffin along the tooth axis, the bones were sectioned into 5 µm slices and stained with H&E for periodontal tissue analysis. The procedure was conducted as previously described.^28^

### Quantitative reverse transcription polymerase chain reaction (RT-qPCR)

RNA was extracted using Trizol, and then reverse transcribed into cDNA using a reverse transcription kit (Yeason, Shanghai, China). Relative expression of the target gene was measured using a 2 × qPCR mix containing SYBGreen (Selleck, Shanghai, China). The primers for the targeted genes were listed as follows: IL-6 (Rat) F: AGTTGCCTTCTTGGGACTGA, R: CCTCCGACTTGTGAAGTGGT; IL-1β (Rat) F: AACCTGCTGGTGTGTGACGTTC, R: CAGCACGAGGCTTTTTTGTTGT; IL-18 (Rat) F: TGCCACCTTTTGACAGTGAG, R: AAGGTCCACGGGAAAGACAC; β-actin (Rat) F: GGAGATTACTGCCCTGGCTCCTA, R: GACTCATCGTACTCCTGCTTGCTG. Arg1 (mouse) F: GACCTGGCCTTTGTTGATGT, R: CCATTCTTCTGGACCTCTGC; α7-nAChR (mouse) F: AGTTTTAACCACCAACATTTGGC, R: TTTTCACTCCGGGGTACTCAG; Gapdh (mouse) F: AATGGATTTGGACGCATTGGT, R: TTTGCACTGGTACGTGTTGAT.

### Statistical analysis

Data are presented as mean ± standard deviation (SD). Statistical significance was determined using Brown-Forsythe ANOVA or Kruskal-Wallis test and sat at p-value < 0.05.

## Results

### VNS improved periodontal clinical index and alleviated alveolar bone loss in periodontitis rats

As our primary objective was to evaluate the therapeutic effects of VNS *in vivo*, we first conducted experiments in a periodontitis rat model. We then performed *in vitro* studies to explore the VNS underlying mechanisms, ensuring that the mechanistic findings were directly associated with the observed *in vivo* outcomes.

**Figure 1:**
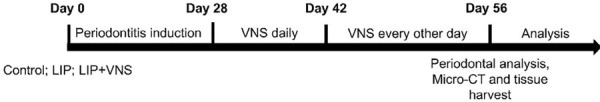
Experimental timeline and procedures

**Figure 2:**
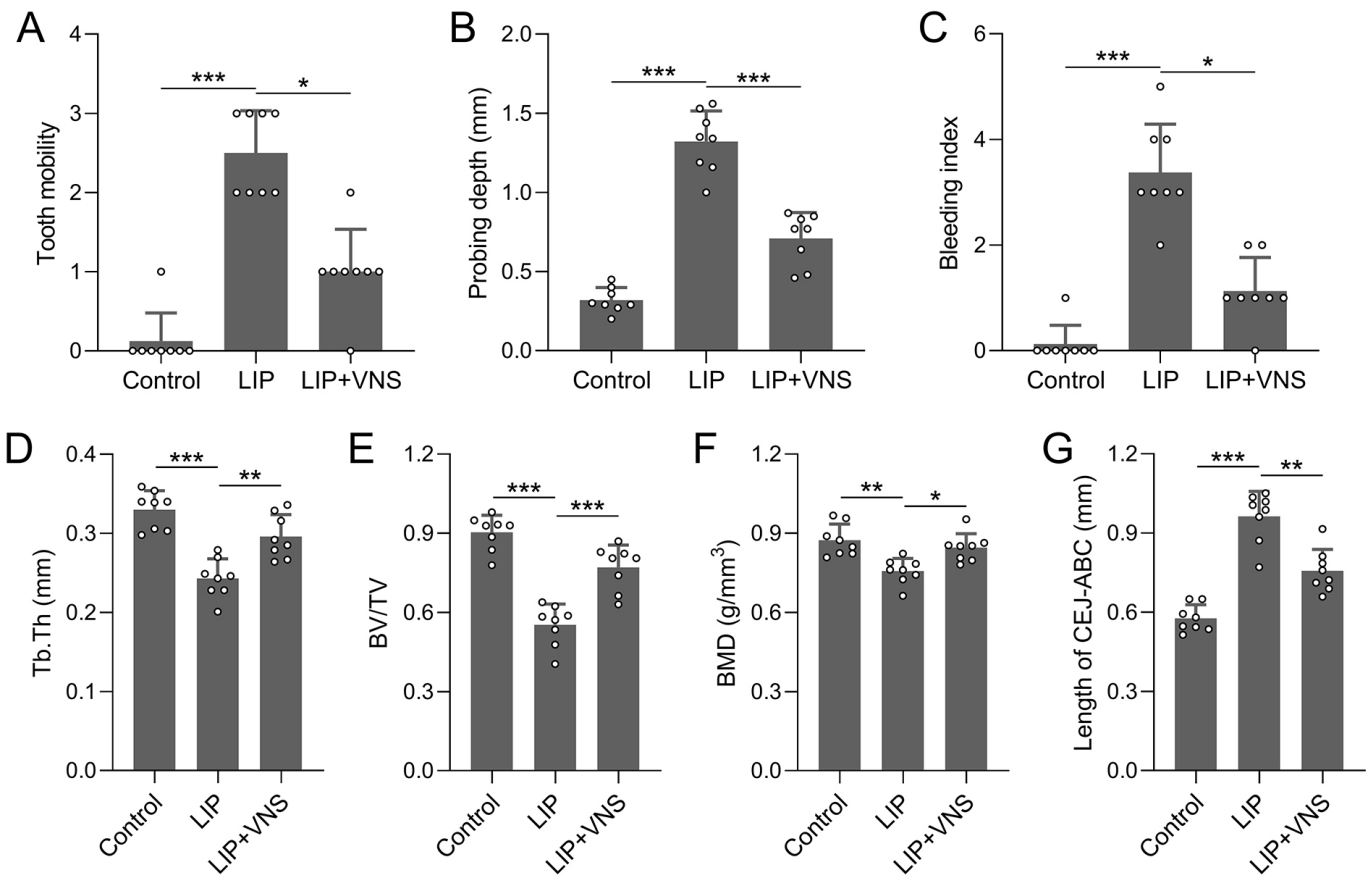
VNS improved periodontal clinical index and alleviated alveolar bone loss in periodontitis rats. Comparisons of tooth mobility (A), probing depth (B), and bleeding index (C) between different groups. Comparisons of Tb. Th (D), BV/TV (E), BMD (F), and the distance from CEJ to ABC (G) were analyzed. n=8 for each group. Data are presented as mean±SD. *p<0.05, **p<0.01, ***p<0.001 by Brown-Forsythe ANOVA test or Kruskal-Wallis test.

Figure 1 summarizes the experimental design, including group allocation, modeling procedures, VNS intervention, and sample collection**.** Initially, periodontal clinical index was evaluated in periodontitis-induced rats after VNS treatment, showing that tooth mobility was higher in the LIP group compared with control, whereas VNS treatment significantly reduced tooth mobility in the LIP+VNS group (Figure 2A). Similarly, probing depth was significantly deeper in the LIP group, but VNS treatment reduced this symptom in the LIP+VNS group (Figure 2B). The bleeding index was higher in the LIP group, and VNS treatment significantly alleviated this effect in the LIP+VNS group (Figure 2C). Analysis of bone parameters revealed that Tb.Th and BV/TV were significantly lower in the LIP group, with VNS treatment improving both parameters (Figure 2D, 2E). BMD also significantly decreased in the LIP group, and VNS treatment significantly enhanced BMD (Figure 2F). Additionally, the length from the cementoenamel junction (CEJ) to the alveolar bone crest (ABC) was greater in the LIP group, but VNS treatment significantly reduced this length (Figure 2G). These results suggest that VNS effectively improves clinical periodontal outcomes and alleviates alveolar bone loss in periodontitis rats.

### VNS alleviated ligature-induced alveolar bone destruction in periodontitis rats

**Figure 3:**
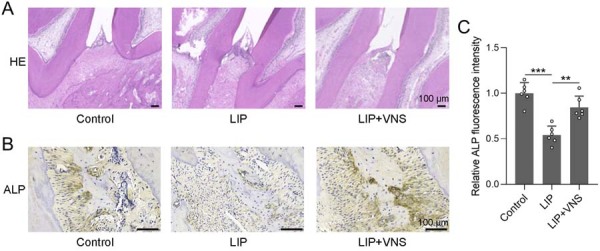
VNS alleviated ligature-induced alveolar bone destruction in periodontitis rats. (A) Representative histological evaluation of HE staining from gingival tissue of rat maxillary first molars. (B) Representative immunohistochemical staining of ALP from gingival tissue of rat maxillary first molars and comparison of relative ALP fluorescence intensity (C). n=6 for each group. Data are presented as mean±SD. **p<0.01, ***p<0.001 by Brown-Forsythe ANOVA test.

Alveolar bone destruction in periodontitis rats was assessed after VNS treatment. Histological analysis of H&E-stained sections showed clear differences in periodontal tissue morphology between the groups (Figure 3A). In controls, the maxillary molar region displayed intact architecture with continuous gingival epithelium, orderly collagen fibers, uniform periodontal ligament width, smooth alveolar bone crest, and minimal inflammatory infiltration. In the LIP group, typical pathological features included apical migration of the junctional epithelium, dense infiltration of lymphocytes and macrophages, widened periodontal ligament space, irregular resorptive alveolar bone surfaces, and marked tissue disruption. These changes were markedly alleviated in the LIP+VNS group which exhibited reduced epithelial migration, less inflammation, more regular ligament structure, smoother bone margins, and partial restoration of tissue integrity, indicating a protective effect of VNS against periodontitis-induced damage. Immunohistochemical staining for ALP showed strong, diffuse expression in the control, markedly diminished levels in the LIP group, and a pronounced recovery in the LIP+VNS group (Figure 3B). Quantitative image analysis used standardized threshold-based segmentation to calculate the mean fluorescence intensity within defined regions of interest (ROI) in the alveolar bone and periodontal ligament (Figure 3C). It revealed that the relative ALP fluorescence intensity in the LIP group was reduced by approximately 50% compared with control (p<0.001), whereas VNS treatment significantly restored ALP expression, achieving a 1.7-fold increase compared with LIP (p<0.01). Collectively, these results indicate that VNS mitigates periodontal tissue destruction and promotes bone regeneration by restoring ALP activity.

### VNS alleviated ligature-induced inflammation in periodontitis rats

**Figure 4:**
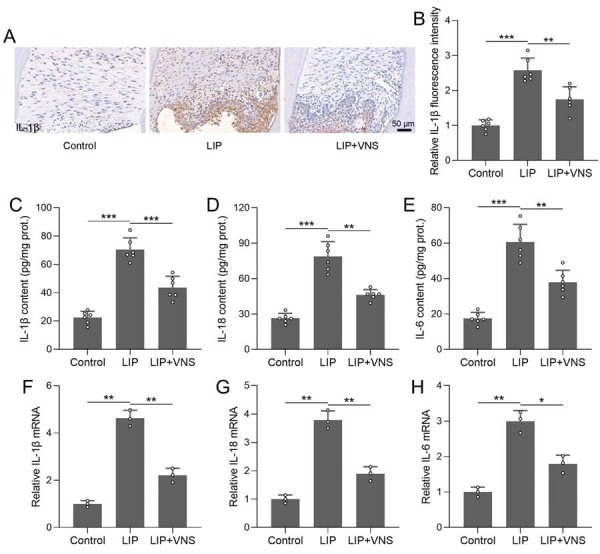
VNS alleviated ligature-induced inflammation in periodontitis rats. (A) Representative immunohistochemical staining of IL-1β from gingival tissue of rat maxillary first molars and comparison of relative IL-1β fluorescence intensity (B). ELISA measured the contents of IL-1β (C), IL-18 (B), and IL-6 (E) in the periodontal tissues of each group. n=6 for each group. mRNA expressions of IL-1β (F), IL-18 (G), and IL-6 (H) in the periodontal tissues were detected by qRT-PCR. n=3 for each group. Data are presented as mean±SD. *p<0.05, **p<0.01, ***p<0.001 by Brown-Forsythe ANOVA test.

VNS treatment effects on ligature-induced inflammation in periodontitis rats was investigated by detecting the IL-1β level. Immunohistochemical analysis of gingival tissue from the maxillary first molars revealed minimal IL-1β immunoreactivity in control, whereas the LIP group exhibited intense and widespread staining, indicating a pronounced inflammatory response. This was markedly attenuated by VNS, as confirmed by a ~2.8-fold increase in relative IL-1β fluorescence intensity in the LIP group (p<0.001) that was reduced by ~40% following VNS (p<0.01) (Figure 4A, 4B). Consistently, ELISA found substantial elevations in IL-1β, IL-18, and IL-6 protein levels in the LIP group compared with control (p<0.001 for all), which were significantly reduced by VNS by approximately 45%, 30%, and 35%, respectively (p<0.01 for all, Figure 4C-E). At the transcriptional level, qRT-PCR analysis showed that mRNA expression of IL-1β, IL-18, and IL-6 was significantly upregulated with about 3-4-fold increase in the LIP group (p<0.01 for all) and downregulated to baseline by VNS (p<0.01 for IL-1β and IL-18; p<0.05 for IL-6, Figure 4F-H). These results indicate that VNS effectively reduces inflammation in periodontitis by suppressing the expression of key pro-inflammatory cytokines.

### VNS activated the cholinergic anti-inflammatory pathway

**Figure 5:**
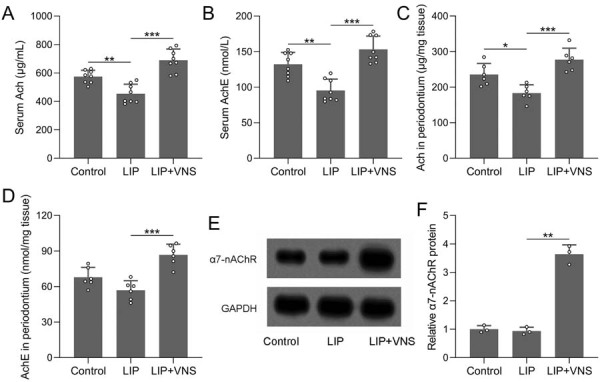
VNS activated the cholinergic anti-inflammatory pathway. ELISA measured the concentrations of Ach (A) and AchE (B) in the serum, and Ach (C) and AchE (D) in the periodontal tissues from each group. n=6 for each group. (E) Western blotting measured the protein expression of α7-nAChR in the periodontal tissues from each group. GAPDH was used as a loading control and the expressions were normalized to control (F). n=3 for each group. Data are presented as mean±SD. *p<0.05, **p<0.01, ***p<0.001 by Brown-Forsythe ANOVA test.

VNS significantly inhibited key pro-inflammatory cytokines in periodontitis rats, suggesting its therapeutic potential in reducing inflammation. To explore the underlying mechanisms, we investigated the activation of the cholinergic anti-inflammatory pathway. Serum Ach levels were significantly higher in the LIP+VNS group compared with the LIP group (Figure 5A). Serum acetylcholinesterase (AchE) levels were also elevated in the LIP+VNS group (Figure 5B). In the periodontal tissues, Ach levels and AchE levels were significantly reduced in the LIP group but increased in the LIP+VNS group (Figure 5C, 5D). Western blot analysis showed increased protein expression of α7-nAChR in the LIP+VNS group compared with the LIP group (Figure 5E, 5F). These findings suggest that VNS activated the cholinergic anti-inflammatory pathway, contributing to reduce periodontitis-induced inflammation.

### Ach activated α7-nAChR and inhibited inflammatory responses in RAW264.7 cells

**Figure 6:**
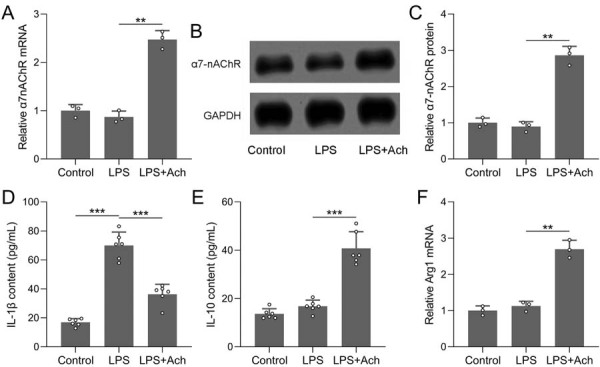
Ach activated α7-nAChR and inhibited inflammatory responses in RAW264.7 cells. (A) qRT-PCR detection of the effect of exogenous ACh intervention on α7-nAChR mRNA expression in RAW264.7 cells. (B) Western blotting measured the protein expression of α7-nAChR in RAW264.7 cells. GAPDH was used as a loading control and the expressions were normalized to control (C). ELISA measured the concentrations of IL-1β (D) and IL-10 (E) in the supernatant of RAW264.7 with exogenous ACh intervention. n=6 for each group. qRT-PCR detection of the effect of exogenous ACh intervention on Arg1 mRNA expression in RAW264.7 cells (F). n=3 for each group. Data are presented as mean±SD. **p<0.01, ***p<0.001 by Brown-Forsythe ANOVA test.

Periodontitis is driven by macrophage-mediated inflammatory responses that promote osteoclast activation and alveolar bone resorption. RAW264.7 cells, a murine macrophage cell line, were therefore selected to model the periodontitis inflammatory component and to investigate the cellular mechanisms underlying the anti-inflammatory effects of VNS. *In vivo*, VNS treatment upregulated α7-nAChR expression in periodontal tissues. Consequently, the *in vitro* effect of α7-nAChR activation mediated by Ach on inhibiting inflammatory responses in RAW264.7 cells was also evaluated. Exogenous ACh treatment increased both mRNA (Figure 6A) and protein expression (Figure 6B, 6C) of α7-nAChR in the LPS+Ach group compared with the LPS group. Ach treatment also reduced secretion of the pro-inflammatory cytokine IL-1β (Figure 6D) and increased the levels of the anti-inflammatory cytokine IL-10 (Figure 6E) in the supernatant of RAW264.7 cells. Additionally, mRNA expression of the anti-inflammatory marker Arg1 was significantly elevated in the LPS+Ach group (Figure 6F). These results suggest that the activation of α7-nAChR by Ach promoted an anti-inflammatory response in RAW264.7 cells.

## Discussion

Our study shows that VNS markedly improves periodontal outcomes in a periodontitis rat model. VNS treatment significantly reduced tooth mobility, probing depth, and bleeding index, and improved bone parameters including trabecular thickness (Tb.Th), bone mineral density (BMD), and bone volume fraction (BV/TV). Histological and immunohistochemical analyses confirmed that VNS alleviated alveolar bone loss, promoted bone regeneration, and suppressed inflammatory cell infiltration. Mechanistically, VNS activated the cholinergic anti-inflammatory pathway, as evidenced by increased ACh levels and α7-nAChR expression, leading to decreased production of pro-inflammatory cytokines (IL-1β, IL-18, IL-6). These findings highlight VNS as a potential non-pharmacological approach that simultaneously mitigates inflammation and supports periodontal tissue repair.

Notably, in our experimental design the ligature was maintained for 4 weeks, which may have allowed partial stabilization of the inflammatory response before endpoint assessment. This extended duration differs from many previous periodontitis studies, in which inflammatory parameters are often evaluated at 14–18 days, a period reported to better capture the peak inflammatory phase before significant resolution occurs. While our primary objective was to model a chronic inflammatory state rather than an acute phase, the choice of a 4-week period could have influenced the cytokine dynamics and tissue responses observed. Future research comparing shorter (14–18 day) and longer ligature durations in otherwise identical protocols will be valuable to determine whether VNS exerts differential effects depending on the inflammation stage.

To further elucidate the cellular mechanisms underlying these *in vivo* observations, we used RAW264.7 macrophages as an *in vitro* model of periodontitis-associated inflammation. This cell line was chosen for its ability to recapitulate macrophage-driven inflammatory responses that drive osteoclast activation and alveolar bone resorption.^29,30^ Building on the *in vivo* finding that VNS upregulated α7-nAChR expression in periodontal tissues, we treated RAW264.7 cells with acetylcholine (ACh), an α7-nAChR agonist, to mimic the cholinergic activation induced by VNS. This approach enabled the direct assessment of anti-inflammatory effects mediated by α7-nAChR signaling at the cellular level, thereby establishing a mechanistic link between the *in vitro* results and the *in vivo* therapeutic outcomes.

Numerous evidence has established that EA stimulation could effectively activate the cholinergic anti-inflammatory pathway, yielding notable anti-inflammatory benefits in a range of inflammatory conditions, including neuroinflammation,^31^ enteritis,^26^ and pancreatitis.^32^ Wang, et al.^31^ (2021) showed that EA treatment significantly upregulated α7-nAChR expression, thereby reducing the production of critical inflammatory mediators. Similarly, Zhang, et al.^32^ (2021) found that EA enhanced the population of α7-nAChR-positive macrophages, consequently diminishing inflammatory responses in a pancreatitis model. Moreover, Lisboa, et al.^33^ (2015) observed that EA application in the oral environment effectively alleviated periodontal inflammation and reduced associated bone damage. While these studies provide valuable mechanistic insights, EA and the direct cervical VNS used in our study differ substantially in methodology. EA delivers electrical stimulation to specific acupoints without surgical exposure of the vagus nerve, whereas our approach involves direct stimulation of the surgically isolated cervical vagus nerve. Both techniques can engage the cholinergic anti-inflammatory pathway; however, differences in stimulation site, degree of invasiveness, and neural recruitment may influence the magnitude, specificity, and clinical translatability of their effects. These distinctions should be considered when comparing outcomes across modalities. Future studies are needed to determine whether less invasive strategies, such as transcutaneous auricular VNS, can reproduce the therapeutic benefits observed with direct cervical stimulation in this model.

The cholinergic anti-inflammatory pathway plays a crucial role in periodontal tissue regulation. Although Ach is predominantly derived from the vagus nerve, its direct functional role in periodontal tissues has yet to be elucidated.^34^ Building on previous research, our study directly examined the effect of VNS on periodontitis. VNS application showed a pronounced activation of the cholinergic anti-inflammatory pathway, as evidenced by increased α7-nAChR expression and elevated ACh levels. This pathway activation suppressed the expression of pro-inflammatory cytokines, including IL-1β, IL-18, and IL-6, and produced significant improvements in clinical periodontal outcomes. Moreover, VNS treatment stimulated bone regeneration and reduced alveolar bone loss, underscoring its dual role in mitigating inflammation and supporting tissue repair. Receptor activator of nuclear factor-κB ligand (RANKL) and osteoprotegerin (OPG), two key regulators of bone remodelling, orchestrate osteoclast differentiation and bone resorption activity via the RANKL/RANK/OPG signalling axis. RANKL, a ligand for receptor activator of nuclear factor-κB, directly promotes osteoclastogenesis by binding to RANK receptors on osteoclast precursors.^35^ In turn, OPG acts as a soluble decoy receptor that competitively binds RANKL, thereby preventing its interaction with RANK and inhibiting osteoclast activation.^36,37^ Elevated RANKL/OPG ratios have been shown to enhance bone resorption, a process strongly associated with alveolar bone loss in patients with periodontitis,^38^ whereas OPG deficiency increases serum RANKL levels and accelerates osteoporosis progression.^35^ Inflammatory microenvironments disrupt bone homeostasis by upregulating RANKL expression while downregulating OPG production.^39^ Consequently, modulation of this axis represents a promising therapeutic strategy for pathological bone resorption.

Our study further validated that VNS not only enhanced clinical periodontal parameters, such as reducing tooth mobility, probing depth, and bleeding index, but also substantially improved Tb.Th and BMD.^33,40^ In conclusion, these results showed that VNS possessed a distinctive ability to simultaneously reduce inflammation and facilitate tissue regeneration. Go, et al.^41^ (2022) highlighted the effectiveness of transcutaneous auricular VNS at 15 Hz, which not only exhibited strong anti-inflammatory properties but also contributed to tissue recovery, underlining its promise as a therapeutic option for managing acute inflammatory conditions. However, the specific molecular mechanisms through which VNS promoted tissue regeneration, as well as its direct relation with reduced inflammation, require further experimental validation.

Our results provide important insights into the molecular mechanism behind VNS-induced anti-inflammatory effects, emphasizing its role in modulating inflammation via the cholinergic anti-inflammatory pathway. Specifically, VNS elevated systemic and local ACh levels and significantly increased α7-nAChR expression.^42^ Our *in vitro* findings, together with previous studies, highlight the role of α7-nAChR in macrophages in inhibiting the transcription of pro-inflammatory cytokines,^31,42^ strongly supporting the hypothesis that VNS reduces cytokines by enhancing cholinergic signaling. This is consistent with earlier studies in which human monocyte-derived macrophages treated with ACh and physostigmine (an AChE inhibitor) under LPS stimulation showed reduced levels of pro-inflammatory cytokines like IL-1β, IL-6, and TNF-α, whereas IL-10 levels remained unchanged.^43^

This study addresses a critical knowledge gap by providing direct experimental evidence that cervical VNS mitigates inflammation and prevents alveolar bone loss in periodontitis by activating the cholinergic anti-inflammatory pathway. In contrast to previous investigations employing pharmacological agents or electroacupuncture, our findings identify VNS as a dual-action therapeutic strategy capable of both modulating inflammatory responses and promoting periodontal tissue regeneration. Future research should focus on optimizing stimulation parameters, determining appropriate treatment durations, and comprehensively evaluating the long-term safety profile of VNS. Particular attention should be given to non-invasive modalities such as transcutaneous auricular VNS, which may offer greater clinical applicability. Moreover, clinical studies are needed to assess its integration with conventional periodontal therapies, explore patient-specific variability in treatment response, and investigate its potential utility in managing comorbid inflammatory conditions. Such efforts will facilitate the translation of these preclinical findings into safe, effective, and clinically relevant neuromodulatory interventions for periodontal disease.

Some study limitations should be highlighted. First, the absence of a control group without induced periodontitis but treated with VNS prevents us from determining whether VNS has baseline effects on healthy periodontal tissues regardless of inflammation. Future studies including such a group would help clarify this point. VNS is known to exert systemic effects beyond its anti-inflammatory actions, including modulation of cardiovascular function (e.g., heart rate and blood pressure regulation) and gastrointestinal motility and secretion.^44,45^ Although these parameters were not systematically measured in our study, routine monitoring did not reveal overt cardiovascular abnormalities or gastrointestinal disturbances. Future work should incorporate dedicated assessments to comprehensively evaluate the systemic safety profile of VNS in the context of periodontitis treatment. While the results clearly showed the anti-inflammatory and bone-regenerative effects of VNS, the exact molecular mechanisms linking cholinergic pathway activation to tissue regeneration are still unclear. Additionally, since the findings are based on an animal model, further research is needed to assess the relevance and effectiveness of VNS for human periodontal disease. These limitations highlight the need for additional studies to fully explore the therapeutic potential of VNS in periodontal disease and its broader clinical applications. We acknowledge that the direct cervical VNS applied in this study is invasive and may limit its clinical applicability. However, non-invasive and translatable alternatives like transcutaneous auricular vagus nerve stimulation (taVNS), which targets the auricular branch of the vagus nerve via the external ear, offer a safer and more practical approach. These methods are currently being evaluated in clinical trials for inflammatory and neurological disorders. Our findings provide mechanistic insight that supports further investigation of non-invasive VNS as a potential therapeutic strategy for periodontitis and other chronic inflammatory conditions.

## Conclusion

In conclusion, VNS improves periodontal health, reduces inflammation, and enhances bone regeneration by activating the cholinergic anti-inflammatory pathway. These findings highlight the potential of VNS as a therapeutic approach for periodontal disease.
